# Motor-Coordination-Dependent Learning, More than Others, Is Impaired in Transgenic Mice Expressing Pseudorabies Virus Immediate-Early Protein IE180

**DOI:** 10.1371/journal.pone.0012123

**Published:** 2010-08-12

**Authors:** Juan C. López-Ramos, Yukiko Tomioka, Masami Morimatsu, Sayo Yamamoto, Kinuyo Ozaki, Etsuro Ono, José M. Delgado-García

**Affiliations:** 1 Neuroscience Division, Pablo de Olavide University, Seville, Spain; 2 Division of Disease Model Innovation, Institute for Genetic Medicine, Hokkaido University, Sapporo, Japan; 3 Laboratory of Biomedicine, Center of Biomedical Research, School of Medical Sciences, Kyushu University, Fukuoka, Japan; L'université Pierre et Marie Curie, France

## Abstract

The cerebellum in transgenic mice expressing pseudorabies virus immediate-early protein IE180 (TgIE96) was substantially diminished in size, and its histoarchitecture was severely disorganized, resulting in severe ataxia. TgIE96 mice can therefore be used as an experimental model to study the involvement of cerebellar circuits in different learning tasks. The performance of three-month-old TgIE96 mice was studied in various behavioral tests, including associative learning (classical eyeblink conditioning), object recognition, spatial orientation (water maze), startle response and prepulse inhibition, and passive avoidance, and compared with that of wild-type mice. Wild-type and TgIE96 mice presented similar reflexively evoked eyeblinks, and acquired classical conditioned eyelid responses with similar learning curves for both trace and delay conditioning paradigms. The two groups of mice also had similar performances during the object recognition test. However, they showed significant differences for the other three tests included in this study. Although both groups of animals were capable of swimming, TgIE96 mice failed to learn the water maze task during the allowed time. The startle response to a severe tone was similar in both control and TgIE96 mice, but the latter were unable to produce a significant prepulse inhibition. TgIE96 mice also presented evident deficits for the proper accomplishment of a passive avoidance test. These results suggest that the cerebellum is not indispensable for the performance of classical eyeblink conditioning and for object recognition tasks, but seems to be necessary for the proper performance of water maze, prepulse inhibition, and passive avoidance tests.

## Introduction

Pseudorabies virus is classified into the genus *Varicellovirus* of the subfamily *Alphaherpesvirinae* [1]. This virus invades and spreads along the trigeminal pathway of neonatal pigs, i.e., the nasal mucosa, trigeminal ganglion, trigeminal nuclei, and their projection areas, such as the cerebellum and thalamus [2], [3]. It causes severe neurological disorders in infected piglets, including nervous signs such as unbalanced stepping, trembling, staggering, and convulsions, and latent infection in surviving pigs. Pseudorabies virus also causes acute and fatal neurological diseases in other domestic and wild animals. In the mouse infection model, this virus induces acute encephalitis similar to that in piglets [4]. Importantly, pseudorabies virus is a highly neurotropic virus that causes neurological symptoms.

Pseudorabies virus expresses a single immediate-early protein, IE180, consisting of 1460 amino acid residues [5]). Pseudorabies virus IE180 exhibits a high degree of homology with the immediate-early protein of other alphaherpesviruses, such as ICP4 of herpes simplex virus type 1 (HSV-1), IE140 of varicella-zoster virus, IE1 of equine herpes virus type 1, and p180 of bovine herpes virus type 1. Pseudorabies virus IE180, like other alphaherpesvirus immediate-early proteins, is known to influence the gene expression of other viruses and mammalian cells [6]–[11].

Based on these findings, we had hypothesized that expression of pseudorabies IE180 would cause the developmental neurological abnormalities in host animals without viral infection and replication. In fact, we previously found that transgenic expression of IE180 in two months old mice caused severe ataxia and cerebellar defects, such as size reduction and disorganized lamination, without any abnormality in other parts of the brain such as hippocampus and cerebral cortex [12]. Further detailed cytological analyses of cerebellum in TgIE96 mice revealed that the expression of pseudorabies virus IE180 caused profound cytoarchitectonic abnormalities involving Purkinje cells, granule cells, molecular layer interneurons, and Bergmann glia, and appeared to affect their cell migration, positioning, cytodifferentiation, dendritogenesis, synaptogenesis, and survival [13]. However, any associated encephalitis observed in the mouse model infected with PRV was not detected in TgIE96. These observations provided important information on causal relationships of cerebellar pathogenicity with cellular defects and PRV IE180. Taken together, these multiple deficits in the cerebellar structures indicate that TgIE96 mice represent a unique experimental model for the study of cerebellar roles in associative learning, as well as in related higher cognitive functions, since severe neural abnormalities presented by these animals are virtually confined to the cerebellum [13].

Accordingly, we used TgIE96 mice here as an experimental model to study the involvement of cerebellar circuits in different learning tasks. Classical conditioning of eyelid responses was carried out in wild-type and TgIE96 mice, using both trace and delay paradigms [14]–[16]. Tones of different durations were used as conditioned stimulus (CS), and an electrical shock presented to the supraorbital nerve was used as unconditioned stimulus (US). Eyelid conditioned responses (CRs) were determined from the electromyographic (EMG) activity of the ipsilateral orbicularis oculi muscle. In addition, the two groups of animals were tested for object discrimination, spatial orientation (water maze), startle response and prepulse inhibition, and passive avoidance. According to the present results, TgIE96 mice have different degrees of learning limitations for the acquisition of new motor abilities depending upon the task, the learning paradigm, and the motor system involved.

## Materials and Methods

### Subjects

Experiments were carried out on male TgIE96 mice and on wild-type littermates having a C57BL/6 genetic background, obtained from the Laboratory of Biomedicine Center of Biomedical Research, Kyushu University (Fukuoka, Japan). Animals were three months old upon their arrival at the Animal House of the Pablo de Olavide University (Seville, Spain), and were kept on a 12-h light/dark cycle with constant temperature (21±1°C) and humidity (50±7%). Animals were allowed *ad libitum* access to commercial mice chow and water. All the experiments were carried out during the light cycle and according to EU (2003/65/CE) and Spanish (BOE 252/34367-91, 2005) guidelines for the use of laboratory animals for chronic behavioral experiments. All experimental protocols were also approved by the Ethics Committee of the Pablo de Olavide University (07/4-20/12/2008).

### Surgical preparation for classical eyeblink conditioning

Under deep anesthesia (Ketamine, 35 mg/kg, and Xylazine, 2 mg/kg, i.p.), animals were implanted with four electrodes in the upper eyelid of the left eye. Electrodes were made of Teflon-insulated, annealed stainless steel wire (50 µm in diameter, A–M Systems, Carlsborg, WA, USA). One pair of electrodes was aimed at the supraorbitary branch of the trigeminal nerve, and served for the presentation of electrical stimuli. The second pair of electrodes was implanted in the ipsilateral orbicularis oculi muscle, and served for recording its electromyographic (EMG) activity. The four electrodes were connected to a 4-pin socket (RS-Amidata, Madrid, Spain) which was fixed with dental cement to the cranial bone. For a week after surgery, animals were kept in independent cages, with free access to food and water, for a proper recovery before the beginning of the experiment. They were maintained in individual cages for the rest of the experiment.

### Classical conditioning procedures

For classical conditioning of eyelid responses, animals were placed individually in a (5 cm ×15 cm ×15 cm) plastic chamber, inside a larger Faraday box (30 cm ×30 cm ×20 cm) to eliminate electrical interferences. Both trace and delay conditioning paradigms were carried out (n = 10 animals per group and paradigm). For this, animals were presented with a tone (6,000 Hz, 70 dB, 20 ms for trace and 250 ms for delay) as a conditioned stimulus (CS), followed 250 ms from CS onset by an electrical stimulation (500 µs, 3× Threshold) as an unconditioned stimulus (US). Intervals between paired CS-US presentations were separated at random by 30±5 s. For habituation and extinction sessions, the CS was presented alone, also at intervals of 30±5 s. A total of two habituation, five conditioning, and four extinction sessions (120 trials each) were presented to each animal across eleven successive days. Only the first fifty trials of each session were analyzed. An additional group of wild-type animals (n = 8) were pseudoconditioned to test the reliability of the task. For pseudoconditioning, unpaired CS and US presentations were carried out for 5 sessions (120 trials each). Pseudoconditioned animals also received two habituation and five extinction sessions as indicated above [17], [18].

The EMG activity of the orbicularis oculi muscle was recorded using differential amplifiers with a bandwidth of 1 Hz to 10 kHz (Grass Technologies, West Warwick, RI, USA). Data were stored directly on a computer through an analog/digital converter (CED 1401 Plus, Cambridge, England), at a sampling frequency of 11–22 kHz and an amplitude resolution of 12 bits. Data were analyzed off-line for quantification of conditioned responses (CRs) with the help of the Signal Average Program (Cambridge Instruments, Cambridge, England). We considered a response to be conditioned when the rectified EMG activity, during the CS-US period, presented the following conditions: i) the EMG activity lasted >10 ms; ii) the EMG was not preceded by any spontaneous activity in the 200 ms preceding CS presentation; iii) the EMG activity was initiated >50 ms after CS onset; and iv) the integrated EMG activity was at least 2.5 times greater than the activity recorded 200 ms before CS presentation [19].

### Object recognition task

For the object recognition task, all the animals (n = 10 per group) were individually habituated to an open field (40 cm ×25 cm ×15 cm), under low illumination conditions, and with no objects, for 5 min. During the training session, two unknown but identical objects (O1 and O2) were placed into the open field, and the animals were allowed to explore them freely for 10 min. The time spent exploring each object and the total approach time were quantified. After each trial, the apparatus and the objects were thoroughly cleaned with 70% ethanol to avoid odor recognition. One hour after the first training, mice were allowed to explore the open field for another 10 min, when one of the two familiar objects (O1 or O2) was replaced by an identical object (O3), and the other (O1 or O2) by a novel object (B1). The time spent exploring each object and the total approach time were quantified again. Within each experimental group, the object O1 was replaced by the new object for half of the animals, whereas object O2 was changed for the other half. The aim was to avoid any issue related to spatial preference associated, or not, with the location of the two objects. Twenty-four hours after the initial training, mice were tested again, with a new object (C1) and an object identical to the old one (B2). The same procedure was carried out 72 h after the initial training (see [20] for details). The attention index (i.e., the percentage of attention) to each object (familiar or new) was expressed as a percentage of the total attention to the two objects exhibited during each session.

### Water maze task

Spatial memory was determined with the help of a modification of the original Morris water maze test [21]. This is a widely used swimming test, which does not constitute a handicap for mice affected by cerebellar ataxia [22]–[24]. Following the protocol described in [25], mice (n = 10 wild-type and 9 TgIE96) were individually trained in a circular pool (100 cm in diameter and 60 cm in height) filled to a depth of 40 cm with water maintained at 25°C. The water was made opaque using a non-toxic white paint. The pool was located in a dimly illuminated room including randomly distributed visual cues. An escape hidden-platform (10 cm in diameter and 1 cm below the water surface) was always placed in the same quadrant of the pool, whilst point of the animal entrance to the pool was changed at random from trial to trial.

The task consisted of three days of probe trials. Each day, the animal was subjected to four trials with an interval of 20 min between trials. Each trial lasted for 60 s (time limit) unless the animal reached the platform in a shorter time. The time taken by the animal to reach the hidden platform was quantified. If an animal failed to find the platform within 60 s, the test was ended and the animal was helped to reach the platform by hand. In either case (whether it reached the platform by itself or not), the mouse was maintained on the platform for 30 additional seconds. The time spent on reaching the platform was recorded on-line, and its percentage with respect to the time limit (60 s) was computed.

### Prepulse inhibition task

The different parameters of the startle reflex and the prepulse inhibition tests were assessed in both wild-type and TgIE96 mice (n = 10 wild-type and 9 TgIE96). Animals were placed individually inside a startle chamber (Cibertec S.A., Madrid, Spain). The startle response was measured using a piezoelectric accelerometer controlled by a computer, using the protocol described elsewhere [24], [26]. The digitized signal was averaged from 25–30 recordings. For training, the mouse was placed in the startle chamber for an acclimation period of 3 min. Baseline responses were averaged after the presentation of 20 sounds (125 dB, 100 ms long). During prepulse inhibition trials, the same 125-dB 100-ms burst was preceded (250 ms) by a prepulse stimulus of 85 dB, lasting for 50 ms. Trials including prepulse stimuli were randomly presented with normal startle stimuli, the final total being 25 of each. The ambient background noise was 70 dB. Four different indexes of prepulse inhibition, related to the response latency, the maximum peak latency, the maximum peak value, and the total response area were recorded and quantified [20]. These indexes were obtained by computing the following formula: ([(startle/prepulse ratio) ×100]/baseline value).

### Passive avoidance task

Experiments were carried out in a passive avoidance device (Ugo Basile, Comerio, Italy). In accordance with procedures described elsewhere [20], each animal (n = 9 per group) was placed in darkness 5 min before training. Then, the mice were placed individually in an illuminated box (10 cm ×13 cm ×15 cm) connected to a dark box of the same size fitted with an electric grid floor, and separated by an automatic door. This door was opened 60 s later. During the acquisition session, entrance of the animal into the dark box was punished by an adequate electric foot-shock (0.5 mA, 1 s). Those animals that did not enter the dark compartment for a first time were excluded from any subsequent experimentation. After 24 h, pre-trained animals were again placed in the illuminated box and observed for up to 5 min (time limit). Animals were re-tested 48 h later. Mice that avoided the dark compartment during the whole time of the experiment were considered to remember the task to the maximum level. The time that the mice took to enter the dark box was noted, and its percentage with respect to the time limit was calculated for each experimental group.

### Data collection and analysis

Data collected from classical conditioning experiments were quantified, through a purpose-designed Excel worksheet, as the percentage of CRs per session – i.e., the proportion of paired CS-US stimulations within a session of 50 presentations that generated an EMG activity satisfying the above-mentioned criteria [19]. Statistical differences between groups were compared across habituation, conditioning, and extinction sessions, using the two-way repeated measures analysis of variance (ANOVA) test, performed with the SPSS 16.0 for Windows package (SPSS Inc, Chicago, IL, USA).

For data collected from object recognition, water maze, prepulse inhibition, and passive avoidance tasks, we compared the statistical differences between groups, using the two-way repeated measures ANOVA test, performed with the same SPSS package. In addition, the Bonferroni *post hoc* test was performed when necessary [20].

## Results

### Experimentally evoked eyeblinks in wild-type and TgIE96 mice

**Figure 1:**
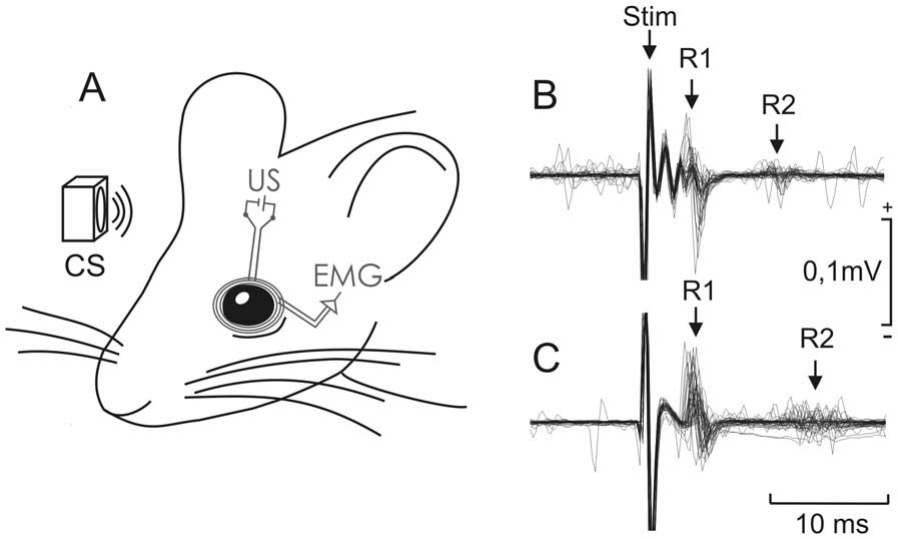
Experimental model used for the classical conditioning of eyelid responses. (*A*) Animals were implanted with bipolar EMG recording electrodes in the left orbicularis oculi (OO) muscle and with stimulating electrodes on the supraorbital nerve. For trace and delay eyeblink conditioning, a tone was used as a CS, lasting 20 ms or 250 ms, respectively. The tone was evoked from a loudspeaker located 30 cm in front of the animal's head. Both CSs were followed 250 ms from their onset by a US consisting of an electrical shock presented to the ipsilateral supraorbital nerve. (*B*, *C*) Superimposed (n = 20) EMG recordings collected from the 1st conditioning session corresponding to a representative animal of the control and TgIE96 groups, respectively. R1 and R2 indicate the presence of the two characteristic EMG components of the blink response evoked by the electrical stimulation (Stim.) of the supraorbital nerve.

In a preliminary series of experiments, we checked whether the neural premotor circuits involved in the generation of eyelid responses functioned normally in both wild-type and TgIE96 mice. The blink reflex can be characterized by measuring the latency of its early (R1) and late (R2) components [27] and the corresponding integrated EMG areas [28]. As illustrated in Fig. 1*B, C*, the eyeblinks evoked by the electrical stimulation of the ipsilateral supraorbital nerve were similar in the two groups of animals. In fact, wild-type (n = 10) animals presented eyeblink values (R1 latency: 4.7±1.2 ms; R2 latency: 10.7±2.3 ms; R1+R2 integrated area: 81.2±12.6 µV × s) similar to those collected from TgIE96 animals (R1 latency: 4.4±1.1 ms; R2 latency: 13.8±4.4 ms; R1+R2 integrated area: 84.3±7.4 µV × s). Moreover, those values were in the range of previous descriptions in mice [17]. Since no significant differences (*P* ≤ 0.814, one-way ANOVA) were observed between reflexively evoked blink responses in the two groups of animals, it was possible to use the classical conditioning of eyeblink responses to test their learning capabilities. Although systematic differences in reflexively evoked eyelid responses would not preclude the possibility of examining whether blink responses may be acquired in an associative task, putative differences in the acquisition process between both groups of animals could be ascribed to the learning process and not to any performance deficit.

### Classical conditioning of eyelid responses using trace and delay paradigms

**Figure 2:**
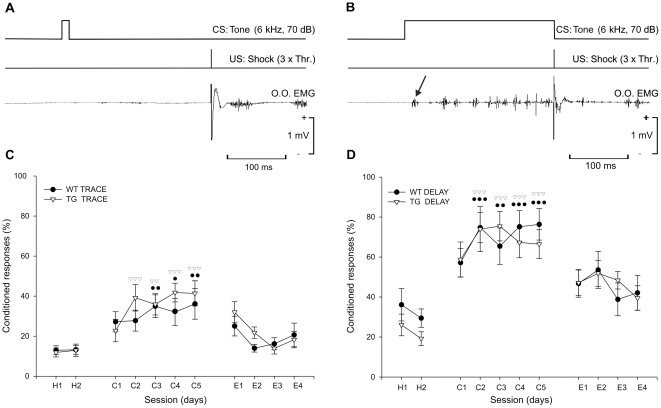
Learning curves collected from the two groups of animals during classical eyeblink conditioning paradigms. (*A, B*) Schematic representation of the trace (*A*) and delay (*B*) conditioning paradigms, illustrating CS and US stimuli presentations and examples of EMG recording obtained during the 1st (for trace) and 5th (for delay) conditioning sessions of representative TgIE96 animals. The arrow in B points to a short-latency alpha response. (*C, D*) Learning curves corresponding to habituation (H1, H2), conditioning (C1–C5), and extinction (E1–E4) sessions obtained during trace (*C*) and delay (*D*) conditioning paradigms, respectively. Data are mean ± SEM of the percentage of CRs from wild-type (WT) and TgIE96 (TG) groups. Two-way ANOVA followed by a *post hoc* analysis showed statistical differences between conditioning and the two habituation sessions of each experimental group, displayed by its own symbols (black circle for WT, and white triangle for TG): one, *P*<0.05; two, *P*<0.01; three, *P*<0.001. In addition, no differences were found between groups for the two different conditioning paradigms.

Figure 2 depicts some raw records (Fig. 2*A, B*) and the mean percentage of CRs across the 5 days of trace (Fig. 2*C*) and delay (Fig. 2*D*) conditioning for the two (wild-type and TgIE96) experimental groups.

During conditioning sessions using a trace paradigm (Fig. 2*A, C*), control animals displayed an acquisition curve characterized by a progressive increase in the percentage of CRs. These animals presented a mean percentage of 27.2±5 (mean ± SEM, n = 10) CRs on the 1st conditioning day, and reached asymptotic values by the 5th conditioning session (36.1±7.6%). Similar values were reached by TgIE96 animals (22.7±5.5% during the 1st conditioning session and 41.4±6.2% during the 5th). The latter (CR values) were more than double the habituation levels. Accordingly, and with respect to trace conditioning, there were no observable significant differences between the percentage of CRs for the two groups [F_(1,15)_ = 0.60; *P* = 0.44]. In contrast, the two-way ANOVA applied to the conditioning data showed a significant difference in the evolution of the percentage of CRs for the two experimental groups with respect to values collected during the habitation sessions [F_(5,55)_ = 9.9; *P*<0.001]. As shown by the Bonferroni *post hoc* test, the wild-type group presented values significantly greater (*P* ≤ 0.01) than those collected during the habituation sessions from the 3rd to the 5th conditioning sessions, whilst the TgIE96 group presented percentages of CRs significantly (*P* ≤ 0.01) different from habituation values from the 2nd to the 5th conditioning sessions. During the extinction process, animals presented a decrease in the percentage of CRs, reaching 20.6±5.8% for the wild-type group, and 18.2±4% for the TgIE96 group by the 4th extinction session. No significant statistical difference could be detected between the two groups during the extinction sessions [F_(1,13)_ = 0.21; *P* = 0.65], suggesting a similar evolution of the percentage of CRs during the extinction process. Moreover, for both experimental groups no statistical difference was found between CR values collected during the 4th extinction session and those corresponding to the habituation session [F_(1,22)_ = 2.5; *P* = 0.12].

For the delay paradigm (Fig. 2*B, D*), wild-type animals (n = 10) presented a mean percentage of 57.2±7.1 CRs in the 1st conditioning session, reaching peak values by the 5th conditioning session (76.3±7.8%). Similar values were reached by TgIE96 animals (58.7±8.7% during the 1st conditioning session and 66.4±7.1% during the 5th). The values were, again, more than the double of the habituation levels. No significant differences in the percentage of CRs for the two groups were observed [F_(1,16)_ = 1.62; *P* = 0.22]. The two-way ANOVA applied to the conditioning data showed a significant difference in the percentage of CRs for the two experimental groups with respect to values collected during the habituation sessions [F_(15,50)_ = 15.03; *P*<0.001]. As shown by the Bonferroni *post hoc* test, the wild-type group presented values significantly higher (*P* ≤ 0.01) than those collected during the habituation sessions from the 2nd to the 5th conditioning sessions, whilst the TgIE96 group presented percentages of CRs significantly (*P* ≤ 0.01) different from habituation values from the 1st to the 5th conditioning sessions. During the extinction process, animals presented a decrease in the percentage of CRs, reaching 53.4±8.8% for the wild-type group and 47.6±8.4% for the TgIE96 group by the 4th extinction session. No statistical difference could be detected between the two groups during extinction sessions [F_(1,13)_ = 0.001; *P* = 0.98], suggesting a similar decay in the percentage of CRs during the extinction process. In addition, no statistical difference was found between CR values collected during the 4th extinction session and those of the habituation session for the two experimental groups [F_(1,20)_ = 3.35; *P* = 0.08].

Finally, the percentage of CRs for the pseudoconditioned animals (n = 8) did not present differences between habituation, conditioning, or extinction sessions [F_(9,79)_ = 1.12; *P* = 0.35; data not illustrated]. This result confirms the reliability of the conditioning protocols used in the present study.

Taken together, these results indicate that, using trace and delay conditioning paradigms, there were no significant differences between wild-type and TgIE96 animals. Interestingly, both groups of animals reached significantly higher values for the delay paradigm than for the trace one, indicating a greater difficulty (i.e., requiring a larger number of paired CS-US presentations) in the latter conditioning paradigm.

### Object recognition

**Figure 3:**
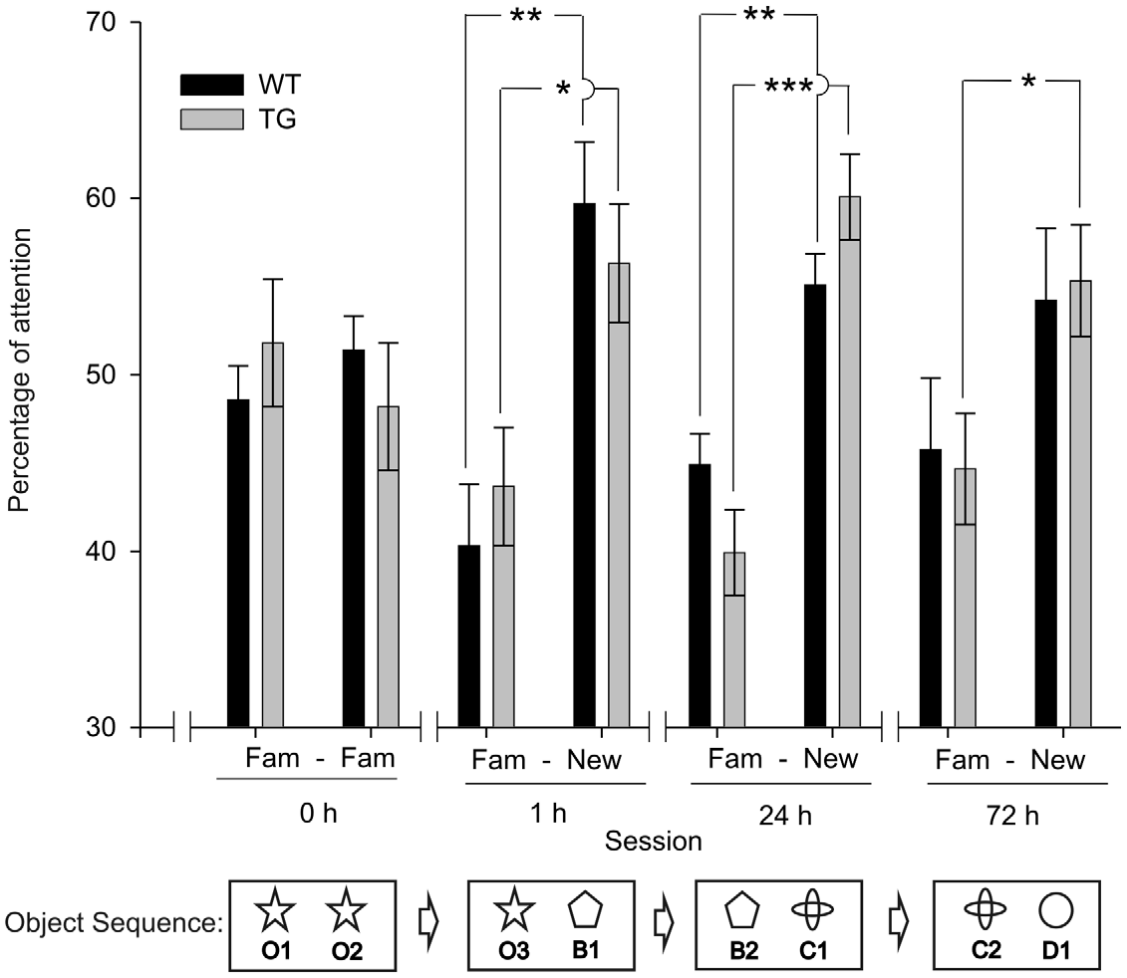
Object recognition task. Representation of the attention devoted to a familiar (Fam) or a novel (New) object exhibited by wild-type (WT) and TgIE96 (TG) groups, during an object recognition task, for the training (0 h) session, and 1, 24, and 72 h afterwards. The object presentation sequence is schematized at the bottom. Values are mean ± SEM of the percentage of the total attention exhibited in each session (see Material and Methods section). *, Statistical differences between percentages of attention, *P*<0.05; **, *P*<0.01; ***, *P*<0.001 (*Post hoc* one-way ANOVA).

In another series of experiments, we compared the learning capabilities of wild-type and TgIE96 animals for object recognition. As illustrated in Fig. 3, during the acquisition period the two groups of animals spent similar amounts of time (about 50%) exploring two identical objects (O1 and O2), indicating no spatial preferences associated with their location. Since the total approach time to the two objects varied from animal to animal, we decided to use percentage of attention (i.e., the number of explorations) as a quantitative index. The percentage of attention was defined as the time spent exploring each object (familiar or new) divided by the time spent exploring both objects and multiplied by 100. The percentage of attention during the acquisition period was similar for objects O1 and O2 for both wild-type [F_(1,18)_ = 1.01; *P* = 0.3] and TgIE96 animals [F_(1,18)_ = 0.5; *P* = 0.4].

During the first choice trial, 1 h after the initial training session, mice were allowed to explore a novel object (B1) and a familiar one (O3). In this case, both wild-type [F_(1,18)_ = 15.5; *P*<0.001] and TgIE96 [F_(1,18)_ = 7.1; *P*<0.01] animals presented a significant increase in the percentage of attention devoted to the novel object (Fig. 3, 1 h session). During the second choice trial, carried out 24 h after the acquisition period (Fig. 3, 24 h session), animals were presented with a novel object (C1) and a familiar one (B2). In this case again, the analysis of variance of the collected data revealed a significant increase in the percentage of attention spent exploring the novel object for both wild-type [F_(1,18)_ = 15.8; *P*<0.001] and TgIE96 animals [F_(1,18)_ = 34.3; *P*<0.001].

During the last choice trial, 72 h after the training session (Fig. 3), mice were allowed to explore a novel object (D1) and a familiar one (C2). Interestingly, in this case both groups of animals explored the novel object for a longer period of time than the familiar one, but the percentage of attention was only significantly higher for the TgIE96 group [F_(1,18)_ = 5.7; *P*<0.02 for TgIE96 animals and F_(1,18)_ = 2.1; P = 0.16 for the wild-type group].

In summary, and as already described for classical eyeblink conditioning, TgIE96 mice performed the object recognition task with the same efficiency as the control group (or ― during the 72 h session ― even better).

### Water maze

**Figure 4:**
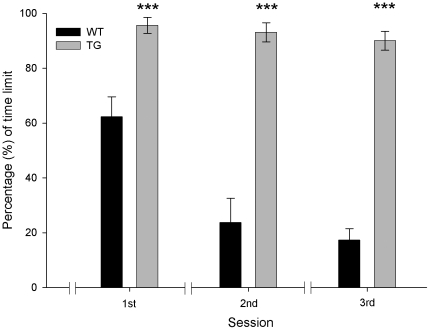
Water maze task. Representation of the time spent by wild-type (WT) and TgIE96 (TG) groups to reach a submerged platform in a water maze task during the three sessions of the experiment. Data represent mean percentage ± SEM of the time limit (60 s = 100%) for each group. **, Statistical differences between groups in a session, *P*<0.01; ***, *P*<0.001 (*Post hoc* one-way ANOVA).

To determine spatial learning and memory abilities of the two groups of animals included in this study we used a modified version of the Morris water maze test. Animals had to find a hidden platform with the help of the visual cues provided. The task consisted of three sessions (three days) each of four trials, separated by 20 min, with each trial lasting a maximum of 60 s. The score obtained in each session was the average of the four scores for each animal. Data were expressed as the percentage (%) of the time limit (60 s). As shown in Fig. 4, wild-type animals performed this test at significantly [F_(1,17)_ = 96.5; *P*<0.001] shorter times than the TgIE96 group. For example, during the first training day, the control group reached the hidden platform at an escape latency (62.3±7.1%) significantly (*P*<0.001) shorter than that for the TgIE96 group (95.6±2.9%). Results collected during the following two tests showed that while wild-type animals improved their performance across training (*P*<0.001 when comparing the 1st vs. the 3rd session), the TgIE96 group did not improve their performance (*P* = 0.23). Taken together, these results indicate that TgIE96 animals presented evident spatial learning and memory deficits as compared with their littermate controls.

### Prepulse inhibition

In accordance with previous descriptions [24] and with present results (see arrow in Fig. 2*B*), both wild-type and TgIE96 mice presented a noticeable alpha response to the tone used as a CS. This also explains the rather high percentage of CRs (alpha responses) collected during habituation sessions, mainly using the delay paradigm (Fig. 2*D*). In consequence, we decided to test the general startle response and the subsequent prepulse inhibition in the two groups of animals. The startle response in the two groups of animals was similar in latency (12.8±0.7 ms for wild-type and 13.6±0.6 ms for TgIE96 mice) and no significantly different in total area (9.92±2.6 against 20.0±7.6 mN/00000 × s).

**Figure 5:**
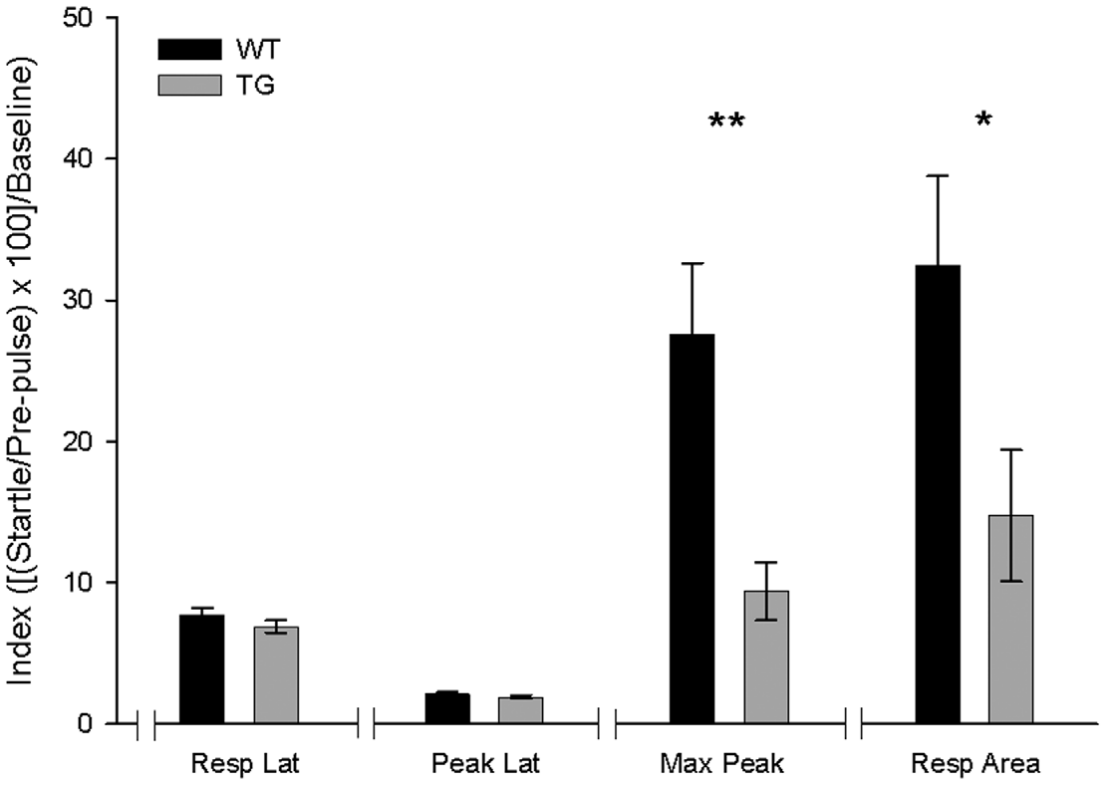
Prepulse inhibition task. Graphical representation of the indexes related to response latency (Resp Lat), maximum peak latency (Peak Lat), maximum peak value (Max Peak), and area (Resp Area) of the responses shown by wild-type (WT) and TgIE96 (TG) groups during a prepulse inhibition task. Values are mean ± SEM of the obtained indexes ([(startle/prepulse ratio) ×100]/baseline value). *, Statistical differences between groups *P*<0.05; **, *P*<0.01 (*Post hoc* one-way ANOVA).

For the sake of homogeneity, and as explained in the Methods section, we used the index [(startle/prepulse ratio) ×100]/baseline to compare data collected from the two groups of animals during the pre-pulse inhibition task. As illustrated in Fig. 5, a one-way ANOVA indicated no significant differences in the indexes related to response [F_(1,17)_ = 1.43; *P* = 0.24] and peak [F_(1,17)_ = 0.9; *P* = 0.3] latencies between wild-type and TgIE96 groups. Nevertheless, the index for maximum peak response was significantly [F_(1,17)_ = 10.1; *P*<0.01] greater for wild-type (performance index = 27.5±5) than for TgIE96 (index = 9.3±2) animals. Moreover, there were significant [F_(1,17)_ = 4.9; *P*<0.05] differences between the two groups with regard to the index for total response area (indexes = 32.4±6.3 for wild-type and 14.7±4.6 for TgIE96 mice). Thus, TgIE96 mice presented some functional deficit in the elaboration of prepulse inhibition when compared with the performance of wild-type animals.

### Passive avoidance

**Figure 6:**
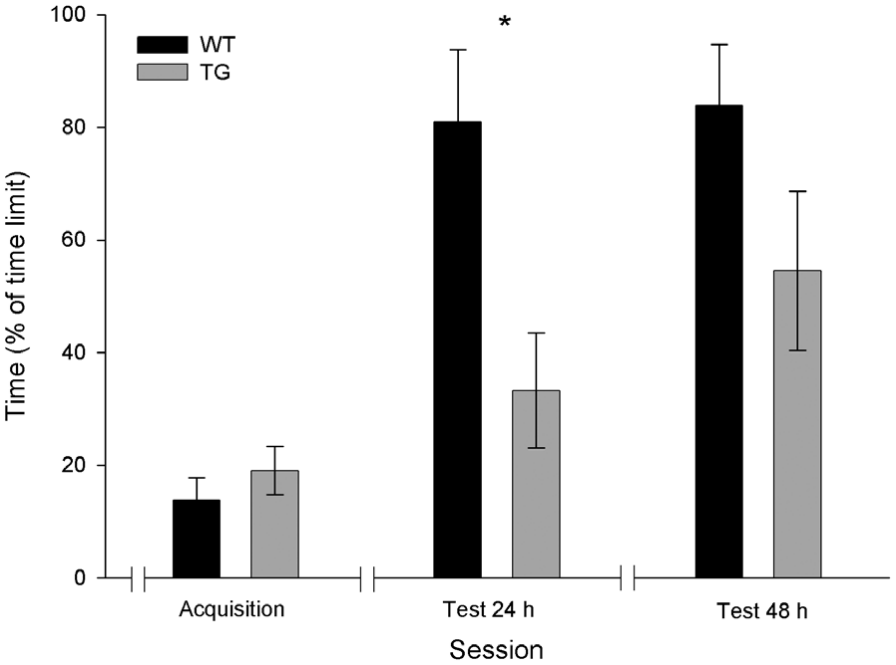
Passive avoidance task. Time spent by wild-type (WT) and TgIE96 (TG) groups to enter the dark area for the Acquisition session, and 24 and 48 h afterwards, during a passive avoidance task. Values are mean ± SEM of the percentage of the time limit (5 min) employed. *, Statistical differences between groups, *P*<0.05 (*Post hoc* one-way ANOVA).

We determined the putative differences in the passive avoidance test for the two experimental groups by measuring the time taken by a mouse to enter the dark compartment after door opening (Fig. 6). For the acquisition session, there was no significant difference between groups in the time spent before entering the dark compartment. The latencies were 41.6±11.8 s (n = 9) for wild-type and 57.1±12.8 s (n = 9) for TgIE96 animals. For comparative purposes, in Fig. 6 is represented the percentage with respect to the maximum time allowed (time limit = 5 min) for the animal to enter the dark compartment (13.9±4.1% for wild-type and 18.8±4.4% for TgIE96 animals). In contrast, for the retention session performed 24 h after the first session, the one-way ANOVA showed a significantly longer time for wild-type animals moving into the dark compartment as compared with the TgIE96 group [F_(1,16)_ = 8.5; *P*<0.05]. As shown in Fig. 6, the wild-type group showed a percentage of retention with respect to the time limit of 80.5±12.6%, whilst the TgIE96 group presented a significantly lower value (33.3±10%). These results suggest a difference in the retention performance of the control vs. the experimental group following the first retention session. In the test carried out 48 h later, although wild-type animals presented longer retention times than the TgIE96 group, no significant differences [F_(1,16)_ = 2.7; *P* = 0.1] were observed between the two groups. Therefore, it can be concluded that in the passive avoidance test, TgIE96 animals present a significant memory deficit as compared with their wild-type littermates.

## Discussion

### General remarks

According to the present results, TgIE96 mice have marked deficits in spatial learning (a function traditionally ascribed to the hippocampus) as shown by their poor performance in the water maze test. In contrast, they performed the object recognition test ― another task involving the participation of hippocampal circuits ― similarly to controls. Moreover, they also presented significant deficits in two tests (prepulse inhibition and passive avoidance) more directly related with selected cerebral (prefrontal cortex) cortical and subcortical (amygdaloid complex) structures. As already reported for Lurcher mice [19], motor performance and learning deficits in TgIE96 mice were more evident for skeletal muscles than for the facial motor system, since the learning curves displayed by these mice during classical conditioning (using both trace and delay paradigms) of eyelid responses were similar to those of controls. These results suggest that cerebellar circuits have a different control for facial muscles than for the skeletal motor system, in coincidence with the particular embryonic origin of the former, a fact already pointed out elsewhere [29]. Finally, the present results indicate that TgIE96 mice represent an excellent experimental model for the study of cerebellar roles in associative learning, as well as in related higher cognitive functions, since neural abnormalities presented by these animals are virtually confined to the cerebellum [16].

### Learning tasks for which TgIE96 mice present capabilities similar to those of their littermate controls

The classical conditioning of eyelid responses allowed us to test the effects of cerebellar damage on the acquisition of this type of associative learning. Neural circuits involved in the generation of reflexively evoked eyeblinks were functionally active in TgIE96 animals generating both components (R1 and R2) of the reflex response following electrical stimulation of the supraorbitary nerve [27], as compared with their littermate controls and with previous studies in Lurcher mutants [19] and wild-type [17] mice. Moreover, TgIE96 mice acquired this associative learning test (using both trace and delay conditioning paradigms) as did controls, indicating that this learning capability was not affected. Recently, an evident deficit for classical eyeblink conditioning has been reported in *pcd* (Purkinje cell degeneration) mice [30]. Since *pcd* mice also present affected inferior olivary neurons and some other neuronal groups located far from the cerebellum (retinal photoreceptors, olfactory mitral cells, and thalamic neurons), the reported deficits cannot be confidently ascribed to cerebellar circuits. As shown in a recent study using classical conditioning of eyelid responses and sumultaneous recordings of multiunitary activity in the red nucleus and in the cerebellar interpositus nucleus [16], cerebellar deficits present in Lurcher mutants produce a decrease in the amplitude of the evoked CR, without affecting their learning curves, a result explaining some discrepancies with earlier reports [14], [15], [30].

As already described in Lurcher mice [19], an evident alpha response to the tone was observed in both TgIE96 and control mice at about 12–15 ms after CS presentation. An alpha response was also noticed in GluRδ2−/− and control mice [31]. Other authors removed the startle response from their analyses [15], [30] to avoid its being quantified together with the other components of the CR. In our case, and following the same criterion, we did not include the alpha component in the computation of CRs. Other studies in genetically manipulated mice indicated that the cerebellum is necessary for the acquisition of classical eyeblink conditioning, mainly when using delay conditioning procedures [32]. It is important to point out that, in mice, learning curves usually present a faster increase in the percentage of CRs as compared with the case of other species, such as rabbits [33] and cats [28], in which we have used similar conditioning protocols. As recently proposed [34], it is still possible that, in mice, the early steps of classical eyeblink conditioning are highly dependent on amygdalar circuits, which could also explain why no evident deficits were observed in TgIE96 mice for this type of associative learning test.

The involvement of cerebellar circuits in object recognition tasks has been shown using functional MRI [35] and magnetoencephalographic [36] recording procedures in humans. In the present study, no significant differences were found between TgIE96 and control mice for this specific discrimination task. As already indicated for classical eyeblink conditioning, it is possible that mice are different in this respect to other species of mammals. Moreover, object recognition tasks seem to involve the specific activation of the hippocampus. This has been shown recently by changes in the strength of hippocampal pyramidal CA3-CA1 synapses during the recognition period in behaving mice [37]. Moreover, mGluR1-rescue mice, which express the metabotropic glutamate receptor-subtype 1 (mGluR1) only in Purkinje cells (PCs) of their cerebellum are incapable of accomplishing an object recognition test [31], indicating that other structures expressing the mGluR1 (probably the hippocampus) are necessary for the accomplishment of this long-term memory test.

### Learning tasks for which TgIE96 mice present significant acquisition and/or memory deficits as compared with their littermate controls

The presence of noticeable deficits in the proper execution of water maze, prepulse inhibition, and passive avoidance tasks in TgIE96 animals raises interesting questions regarding cerebellar contributions to those different learning tasks. Indeed, these three learning tests involve the participation of many different cortical (prefrontal, parietal) and subcortical (basal ganglia, thalamus, amygdala) structures [38]. Assuming that neural deficits present in TgIE96 mice are restricted to cerebellar circuits [16], we could propose that the cerebellum is involved not only in motor control, but also in more-general functions, such as spatial orientation, emotional displays, and cognitive processes.

TgIE96 animals presented a noticeable learning and memory deficit for acquisition and retention of the Morris water maze task, a fact that cannot be ascribed to their ataxic syndrome, since they were capable of swimming like their littermate controls, as also observed in other cerebellar-deficient mice [22]–[24], [39]. For example, Lurcher mice (presenting an overall degeneration of cerebellar Purkinje and granular cells) have a significant orientation deficit in the Morris water maze test [24], but still retain some capability for this type of motor and orientation learning [40]–[44], an attribute not found in TgIE96 mice. Other types of genetically manipulated mice (for example, TAG-1 deficient and P311 knockout mice) present cognitive impairments in the Morris water maze, but in these cases, other cerebral structures (such as the hippocampus, the entorhinal cortex, and the olfactory bulbs) seem to be affected besides the cerebellum [45], [46]. In summary, according to the present results, the cerebellum seems to be involved in spatial learning and/or spatial orientation as well as in visuomotor [47] and oculomotor [48] coordination.

Although the startle response to a severe tone [49] presented no significant differences in both wild-type and TgIE96 mice, we found a reduction in the ability to produce prepulse inhibition in the latter group of animals. There are few studies involving the cerebellum and the startle response in mice, and even fewer relating cerebellar lesions and prepulse inhibition of the startle response [24]. Nevertheless, it has been shown that the medial cerebellum is necessary to evoke the startle response, mainly in long-term habituation [50], [51], but the inferior olive is not [52]. On the other hand, mice with hypoplasia of the anterior cerebellar vermis have a normal startle response, as well as its prepulse inhibition [53]. In contrast, Lurcher mice have a normal startle response, but an evident deficit in prepulse inhibition [24]. Apparently, the impaired maturation of cortical cerebellar (and cerebral) dendritic spines also prevents a proper prepulse inhibition in mice [54]. Since brainstem pontine structures have been proposed as mediating the prepulse inhibition of the startle response [55], the lack of prepulse inhibition observed in TgIE96 (present experiments) and in Lurcher [24] mice suggests a deficit in its sensory integration at the level of the brainstem or a functional deficit in the projection to the reticular formation from cerebellar structures (i.e., deep nuclei) involved in the startle response.

Neural mechanisms involved in emotional reactivity, such as those mediating passive avoidance tasks, are classically assumed to take place in amygdalar circuits [56]–[59]. In contrast, there is scarce information regarding cerebellar involvement in active or passive avoidance tests [59]–[61]. According to the present results, alteration of cerebellar circuits is able to disrupt the animal's performance in a passive avoidance test, a deficit also reported in Lurcher mice using a modified version of the passive avoidance test [62]. The involvement of the cerebellum in active avoidance tasks has also been reported in rats [59]. In contrast, Rora (sg) mutant mice with mild granule cell degeneration and Grid2 (ho) mice with more-severe granule cell degeneration together with Purkinje cell atrophy did not differ from controls in a passive avoidance learning task [63]. Although the involvement of the cerebellum in cognition is still a matter of debate (for example, see [64]), present results suggest that this structure could be involved in some types of discriminative learning, such as the passive avoidance task.

Finally, it can be indicated that in order to properly determine the contribution of cerebellar structures not only to the improvement of motor skills, but also to the acquisition of new motor and cognitive abilities, it will be necessary to use many different associative and non-associative learning tasks as shown by the present study. In addition, it has been recently reported that the cerebellum contributed to the ongoing functional states [65] of the neural systems controlling motor performance [66] with a modulating/reinforcing role, as shown during classical eyeblink conditioning in behaving mice [16] and cats [67].
